# Xanthones Content in *Swertia multicaulis* D. Don from Nepal

**DOI:** 10.3390/molecules23051067

**Published:** 2018-05-03

**Authors:** Binu Timsina, Pavel Kindlmann, Maan B. Rokaya, Naděžda Vrchotová, Jan Tříska, Štěpán Horník, Jan Sýkora

**Affiliations:** 1 Institute for Environmental Studies, Faculty of Science, Charles University, Benátská 2, 128 01 Prague, Czech Republic; binu.timsina@gmail.com (B.T.); kindlmann.p@czechglobe.cz (P.K.); 2 Department of Biodiversity Research, Global Change Research Institute, Czech Academy of Sciences, Bělidla 986/4a, 603 00 Brno, Czech Republic; rokayamaan@gmail.com; 3 Institute of Botany, Czech Academy of Sciences, Zámek 1, 252 43 Průhonice, Czech Republic; 4 Laboratory of Metabolomics and Isotopic Analyses, Global Change Research Institute, Czech Academy of Sciences, Bělidla 986/4a, 603 00 Brno, Czech Republic; vrchotova.n@czechglobe.cz; 5 Institute of Chemical Process Fundamentals, Czech Academy of Sciences, Rozvojová 135, 165 02 Prague, Czech Republic; hornik@icpf.cas.cz (Š.H.); sykora@icpf.cas.cz (J.S.)

**Keywords:** *Swertia*, Gentianaceae, HPLC, LC–MS, NMR, phytochemistry, secondary metabolites

## Abstract

The medicinal plant *Swertia multicaulis* D. Don was collected in Rasuwa District (Nepal) and the xanthone content of its ethyl acetate extracts was studied. The total amount of xanthones in *S. multicaulis* determined by HPLC reaches almost 13 g of xanthones per 1 kg of dry matter. The identification of xanthones in *S. multicaulis* was achieved by a combination of HPLC, LC–MS and LC–NMR. The final assignment of the individual chemical structures was provided by NMR, supported by preparative HPLC. In eight chromatographic peaks, four major xanthones were identified—1,3-dihydroxy-5,8-dimethoxyxanthone, 1-hydroxy-3,5,8-trimethoxyxanthone, bellidifolin (1,5,8-tri-hydroxy-3-methoxyxanthone), and decussatin (1-hydroxy-3,7,8-trimethoxyxanthone).

## 1. Introduction

*Swertia* is one of the 87 genera of the Gentianaceae family and comprises about 170 species that are widespread throughout the world [1]. *Swertia* has been used in Indian, Tibetan and Nepalese folk medicine for centuries. The therapeutic effects are mainly associated with high levels of xanthones. The unique properties of xanthones are used against a wide range of diseases, including malaria, diabetes, cancer, liver diseases, and others [2]. Numerous publications have described the content of these compounds in various species of *Swertia* [3,4,5,6,7,8,9]. As individual xanthones vary in polarity, the content of xanthones in extracts depends significantly on the solvent used. Wolfender et al. [10] extracted and analysed *S. calycina.* Starting with 61 g of dry matter, and 3 g of compounds were obtained by extraction with dichloromethane. By extraction with methanol, almost 8 g of material was obtained. In addition to xanthones, other substances (e.g., quinones) were identified. The xanthones swertianolin and bellidifolin were identified in six species of *Swertia* (*S. japonica*, *S. pseudochinensis*, *S. delavayi*, *S. decor*, *S. binchuangensis*, and *S. punicea*) [3]. Decussatin has been recorded as occurring in *S. calycina* [10], *S. decussata* [11], and *S. mussotii* [12]. The review of Brahmachari et al. [1] relates decussatin’s reported presence also in *S. patens*, *S. hookeri*, *S. petiolata*, *S. perfoliata*, *S. chirata*, *S. purpurascens*, *S. milensis*, *S. paniculata*, *S. punicea*, *S. lawii*, *S. nervosa*, *S. racemosa*, *S. perennis*, *S. dialata*, and *S. gracilescens.* However, the highest content of these compounds has been recorded in *S. japonica*, where the content of swertianolin ranged from 2.5 to 5.8 g/kg of dry matter and that of bellidifolin from 4.2 to 8.5 g/kg [3]. One kilogram of whole plant *S. mussotii* can provide 16 mg of methylswertianin, 42 mg of swerchirin, and 22 mg of decussatin [12]. In the study by Khanal et al. [13], the content of the xanthone mangiferin ranged from 150 to 220 mg per 1 kg of dry matter in *S. chyrayita, S. angustifolia*, *S. paniculata*, *S. racemosa, S. nervosa*, *S. ciliata*, and *S. dilatata*. Besides xanthones, information about iridoids, terpenoids, alkaloids, and the content of other substances in various species of *Swertia* can be found in several reviews [1,14,15]. However, some *Swertia* species still remain unexplored. There is no information, for example, on the secondary metabolites of *S. multicaulis* D. Don. This plant is used in traditional Nepalese medicine for treating wounds, fevers, coughs, colds, stomach pains, and constipation, as well as for expelling roundworms from the alimentary canal [16,17,18,19,20,21]. The plant grows at altitudes of 4000–4900 m a.s.l. in India, Bhutan, south-east Tibet, and Nepal. Its Nepalese name is sarma guru and in English it is known as chiretta. In this paper, we present the identification of major xanthones in ethyl acetate extracts of this plant. To the best of our knowledge, this is the first study of this kind regarding *S. multicaulis*.

## 2. Results and Discussion

**Figure 1:**
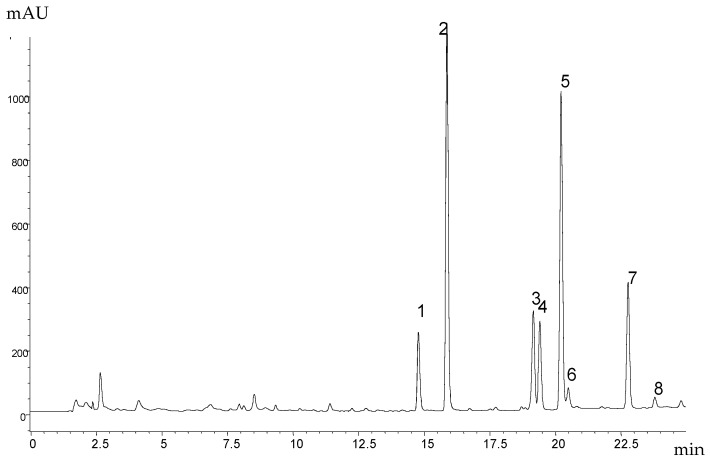
HPLC analysis of the ethyl acetate extract of the above-ground parts of *S. multicaulis* (sample SM1); monitored at 315 nm.

**Figure 2:**
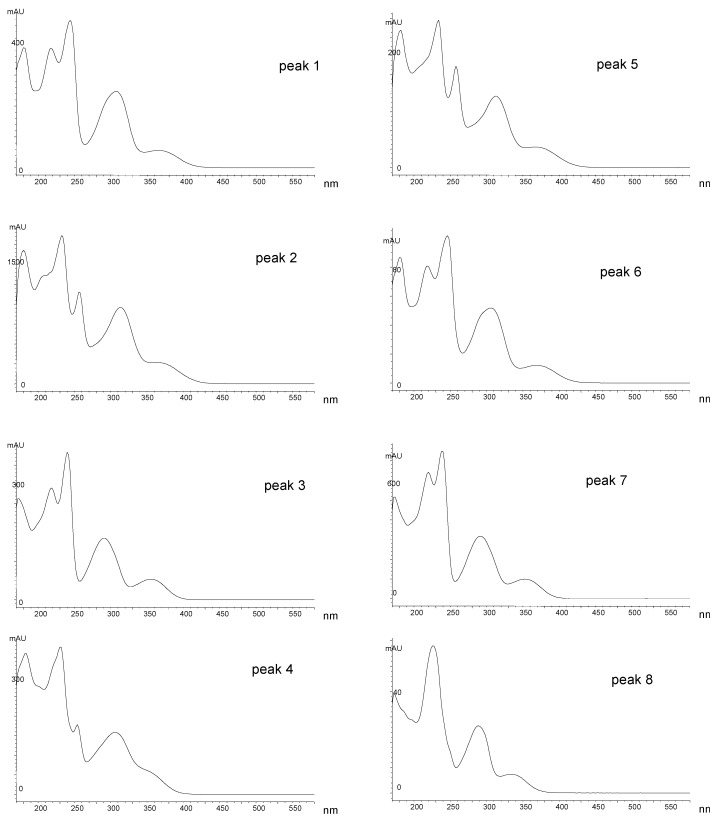
Overview of UV spectra of individual peaks obtained using high-performance liquid chromatography (labelling of peaks, see Figure 1).

The above-ground, non-flowering parts of *S. multicaulis* D. Don were extracted by ethyl acetate. The extract was transferred into methanol (for details see Materials and Methods) and submitted to analysis by high-performance liquid chromatography (HPLC). The HPLC analysis was monitored by UV spectroscopy and mass spectrometry (MS). Spectral analysis revealed that the dominant compounds have similar UV and MS spectra and probably belong to the xanthone family. The chromatographic profile of the SM1 sample is shown in Figure 1 and an overview of the UV spectra of individual peaks is shown in Figure 2.

Individual xanthones differ in the number and position of hydroxyl and methoxy groups. Therefore, the identification of individual compounds is very difficult and usually requires a combination of several instrumentation techniques. MS spectra can be used e.g., only for determination of the number of methoxy groups in the molecule. Molecular ions of the individual peaks obtained by liquid chromatography–mass spectrometry (LC–MS) in “Full Scan” mode are listed in Table 1.

**Figure 3:**
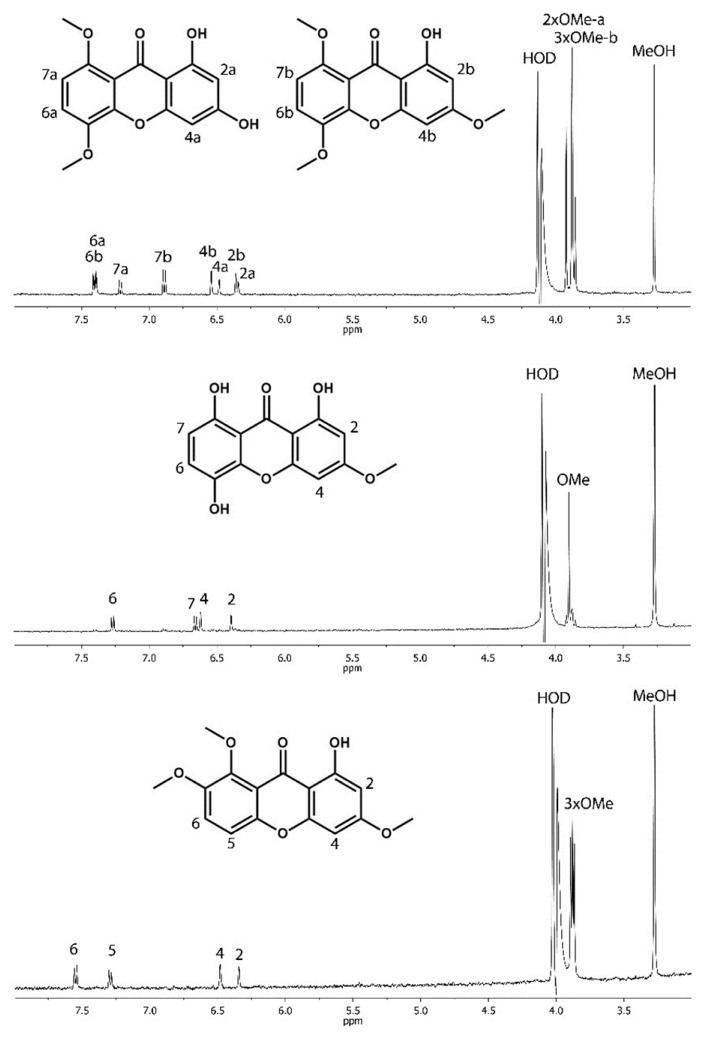
Liquid chromatography and nuclear magnetic resonance (NMR) spectroscopy traces. ^1^H-NMR spectra of peaks 3 and 4 showing 1,3-dihydroxy-5,8-dimethoxyxanthone and 1-hydroxy-3,5,8-trimethoxyxanthone, respectively (**top**). Peak 5 is bellidifolin (**middle**) and Peak 7 is decussatin (**bottom**).

The final peak identification was attempted by an analysis combining liquid chromatography (LC) and nuclear magnetic resonance (NMR) spectroscopy (LC–NMR) which provides authentic spectra of chromatographic peaks. LC–NMR analysis was performed solely in stop-flow mode due to low concentrations of the compounds in individual chromatographic peaks. The low concentrations prevented the application of two-dimensional NMR experiments, as only ^1^H-NMR spectra could be collected within a reasonable period of time. Nevertheless, the LC–NMR analysis proved that all chromatographic peaks contained compounds belonging to the xanthone family (Figure 3). The analysed compounds differed only in the positions of the hydroxyl groups and/or numbers of methoxy groups. Under the given chromatographic conditions, the more polar compounds having fewer methoxy substituents eluted prior to the less polar compounds. The final structure assignment could be accomplished only by comparing individual ^1^H-NMR spectra with data in the literature. For this purpose, individual chromatographic peaks were collected in repeated HPLC runs, evaporated and then dissolved in corresponding deuterated solvents (CD_3_OD, CDCl_3_, DMSO). Nevertheless, the overall concentrations remained low.

The combined fraction of chromatographic peaks 1 and 2 provided only a diluted mixture of polar xanthones, usually with no or just one methoxy group. The fraction contained more than four compounds. No match was found with data in the literature. The corresponding ^1^H-NMR spectra and proposed structures can be found in the Supplementary Material. Chromatographic peaks 3 and 4 were inseparable under the HPLC conditions used. Despite co-elution, the resulting fraction contained only two compounds. These were identified as 1,3-dihydroxy-5,8-dimethoxyxanthone (Peak 3) [22] and 1-hydroxy-3,5,8-trimethoxyxanthone (Peak 4) [23]. The final assignment of the chromatographic peaks was made by comparing LC–NMR and LC–MS data. Chromatographic peaks 5 and 7 each contained one dominant compound. The spectrum of peak 5 corresponded well with bellidifolin (1,5,8-trihydroxy-3-methoxyxanthone) [24], while peak 7 contained decussatin (1-hydroxy-3,7,8-trimethoxyxanthone) [10]. Only the concentration of decussatin allowed the measurement of the ^13^C-NMR spectrum. The collected fractions of peaks 6 and 8 were below the detection limit of the NMR spectrometer used. The resulting structures assigned to LC–NMR data are depicted in Figure 3. 

Using a calibration curve built for isogentisin, the xanthone content was quantified. The content of individual chromatographic peaks found is summarized in Table 2. The average total content of xanthone found in the above-ground, non-flowering part of the plant was almost 13 g per 1 kg of dry matter.

The xanthone content found in *S. multicaulis* is quite high when compared to other *Swertia* species. From this point of view, this plant deserves further attention, and detailed screening of the biological activity of its extracts should be performed in future. On the other hand, the growing demand for medicinal plants and the destructive way in which they are collected threaten the natural habitats of these plants. Nepalese medicinal plants are no exception [25]. Therefore, it is important to identify plant species containing the same or similar compounds to substitute the traditional plants and to decrease the harvesting pressure put on these plants. Regarding xanthones, similar compounds were identified e.g., in *Garcinia mangostana* [2]. In this plant, the xanthone derivatives bear besides hydroxy- and methoxy groups mainly isoprenyl groups. *G. mangostana* could therefore provide an alternative to endangered *Swertia* species as source of xanthones.

## 3. Materials and Methods 

### 3.1. Plant Materials and Extraction

*S. multicaulis* D. Don is a perennial plant growing 8–12 cm tall and with a taproot 5–10 mm in diameter. The leaves are mostly basal, occurring in a rosette, base connate. The leaf blade is spatulate to oblong-spatulate, 1.5–3.5 cm × 5–12 mm, base narrowed, margin scabrous, apex obtuse to rounded. Stem leaves occur in 1 or 2 pairs, sessile, bract-like, elliptic, 7–10 × 3–5 mm in size, both ends obtuse, midvein distinct. Inflorescences are raceme-like or umbel-like. Flowers are numerous and small. Calyx lobes are lanceolate to oblong, 5–7 mm in size. Corollas are pale blue to purple, 1.5–2.2 cm in diameter. Fruits are in ovoid–ellipsoid capsules, 1.2–1.5 cm. Seeds are brown, subglobose, almost smooth. Flowering and fruiting time is June–September [26].

Above-ground parts of four flowering plants of *S. multicaulis* D. Don were collected during August 2012 from the Surya Kund area (at 4665 m a.s.l.) in central Nepal’s Rasuwa District. They were air-dried in the shade and stored in muslin bags until chemically analysed. Samples were marked SM1–SM4. The thoroughly milled material was extracted using ethyl acetate at 50 °C for 40 min. Zero point twenty five grams of milled plant material was extracted with 3 mL of ethyl acetate. After centrifugation, the sediment was washed twice with 1 mL of ethyl acetate. Supernatants were pooled, the ethyl acetate was evaporated off, and the residue was then dissolved in methanol. Extracts were stored at −18 °C until analysed using HPLC.

### 3.2. HPLC and LC–MS Measurements

HPLC was performed using an HP 1050 device (Hewlett-Packard, Palo Alto, CA, USA), an Agilent G1315B DAD detector (Agilent, Santa Clara, CA, USA), and a Phenomenex Luna C18 (2), 3 µm, 2 × 150 mm column (Phenomenex, Torrance, CA, USA). Mobile phases (acetonitrile–water–*o*-phosphoric acid) were as follows: mobile phase A—5% acetonitrile + 0.1% *o*-phosphoric acid; mobile phase B—80% acetonitrile + 0.1% *o*-phosphoric acid. The gradient was 20% B–80% B for 20 min and then 80% B–100% B for 5 min. The flow rate was 0.25 mL/min. The injected volume was 5 µL. The column temperature was 25 °C. The separation was monitored at 315 nm. 

HPLC was used also for compound quantification. Xanthone content was calculated using a calibration curve built for isogentisin (PhytoLab GmbH & Co. KG, Vestenbergsgreuth, Germany). The method was validated. The detection limit of isogentisin was estimated at 0.11 µg/mL and the quantification limit at 0.36 µg/mL. 

LC–MS was performed using an LCQ Accela Fleet device (Thermo Fisher Scientific, San Jose, CA, USA) utilizing atmospheric pressure chemical ionization (APCI). The same column as in the HPLC analysis was used (i.e., Phenomenex Luna C18 (2), 3 µm, 2 × 150 mm). Formic acid was used instead of *o*-phosphoric acid. The column and separation methods were the same as in the HPLC. APCI capillary temperature was 275 °C, APCI vaporizer temperature 450 °C, sheath gas flow 50 L/min, auxiliary gas flow 5 L/min, source voltage 6 kV, source current 5 μA, and capillary voltage −4 V.

### 3.3. LC–NMR Measurement and Preparation of Xanthones

A commercial HPLC system (Dionex UltiMate 3000, Thermo Fisher Scientific) with a 4.6 × 250 mm HPLC column (Luna C18 (2), Phenomenex, 5 μm, 100 Å pore size) was employed. The separation was done by gradient elution using an acetonitrile–water system (starting at 50% acetonitrile, increasing to 80% acetonitrile over 25 min and then to 90% acetonitrile over 35 min) and monitored at 315 nm. The flow rate was 0.5 mL/min. For ^1^H-NMR observations, a Varian INOVA 500 MHz spectrometer equipped with HCN triple resonance (60 µL active volume) micro-flow probe was used. Standard NMR software (VnmrJ 4.2) was used. The separation and NMR detection were conducted at ambient temperature (22 °C). Detailed analysis of chromatographic peaks was performed in the stop-flow mode. Depending on the particular concentration, the ^1^H-NMR spectra were accumulated over at least 128 scans (acquisition time 2 s, relaxation delay 1 s). The WET (water suppression enhanced through T1 effects) multiple frequency solvent suppression method was employed in the measurement. The ^1^H-NMR spectrum was referenced to the signal of acetonitrile (δ = 2.00 ppm). 

The same HPLC instrument was employed for the preparation of xanthones. This involved multiple 50 μL injections of the concentrated methanol solution into HPLC and collection of individual chromatographic peaks. The individual fractions were evaporated and each dissolved in an appropriate deuterated solvent (CD_3_OD, DMSO-*d*_6_) in order to enable comparison with the literature data.

### 3.4. Determination of Xanthones Using NMR

The final NMR data were collected using an Inova500 NMR spectrometer (Varian, Palo Alto, CA, USA) operating at 499.9 MHz for ^1^H and 125.7 for ^13^C. The ^1^H, ^13^C, COSY, HSQC, and HMBC spectra were used for structure elucidation in the case of decussatin (Peak 7). The collected sample of decussatin was measured in CD_3_OD and ^1^H- and ^13^C-NMR spectra were referenced to the line of the solvent (CD_3_OD; δ = 3.31 ppm and δ = 49.00 ppm, respectively). The rest of the trapped chromatographic peaks were dissolved in deuterated solvent corresponding to the literature (CD_3_OD, CDCl_3_, or DMSO) and only ^1^H-NMR spectra were collected.

NMR data for 1,3-dihydroxy-5,8-dimethoxyxanthone (Peak 3) are as follows: ^1^H-NMR (CDCl_3_, ppm) δ: 13.13 (s, 1H, OH), 7.37 (d, 1H, *J* = 9.1 Hz), 7.16 (d, 1H, *J* = 9.1 Hz), 6.37 (d, 1H, *J* = 2.3 Hz), 6.33 (d, 1H, *J* = 2.3 Hz), 5.94 (s, 1H, OH), 4.04 (s, 3H), 3.89 (s, 3H).

NMR data for 1-hydroxy-3,5,8-trimethoxyxanthone (Peak 4) are as follows: ^1^H-NMR (CDCl_3_, ppm) δ: 13.20 (s, 1H, OH), 7.20 (d, 1H, *J* = 9.1 Hz), 6.72 (d, 1H, *J* = 9.1 Hz), 6.50 (d, 1H, *J* = 2.3 Hz), 6.33 (d, 1H, *J* = 2.3 Hz), 3.98 (s, 3H), 3.98 (s, 3H), 3.88 (s, 3H). 

NMR data for 1,5,8-trihydroxy-3-methoxyxanthone (bellidifolin, Peak 5) are as follows: ^1^H-NMR (DMSO-*d*_6_, ppm) δ: 11.92(s, 1H, OH), 11.09 (s, 1H, OH), 9.70 (s, 1H, OH), 7.27 (d, 1H, *J* = 8.8 Hz), 6.66 (d, 1H, *J* = 8.8 Hz), 6.63 (d, 1H, *J* = 2.3 Hz), 6.42 (d, 1H, *J* = 2.3 Hz), 3.91 (s, 3H).

NMR data for 1-hydroxy-3,7,8-trimethoxyxanthone (decussatin, Peak 7) are as follows: ^1^H-NMR (CD_3_OD, ppm) δ: 7.56 (d, 1H, *J* = 9.3 Hz), 7.28 (d, 1H, *J* = 9.3 Hz), 6.46 (d, 1H, *J* = 2.3 Hz), 6.31 (d, 1H, *J* = 2.3 Hz), 3.93 (s, 3H), 3.93 (s, 3H), 3.89 (s, 3H). ^13^C-NMR (CD_3_OD, ppm) δ: 182.62 (s), 168.32 (s), 164.79 (s), 158.77 (s), 152.18 (s), 150.80 (s), 149.49 (s), 121.97 (d), 116.45 (s), 114.15 (d), 104.84 (s), 97.95 (d), 92.96 (d), 62.03 (q), 57.40 (q), 56.47 (q).

## 4. Conclusions

*S. multicaulis* D. Don known from Nepalese folk medicine was found very rich in xanthone content. The xanthone content was quantified and found to be close to 13 g per 1 kg of dry matter. Using HPLC, MS, and NMR spectroscopy supported by preparative HPLC, four major xanthones were identified–namely bellidifolin, decussatin, 1,3-dihydroxy-5,8-dimethoxyxanthone and 1-hydroxy-3,5,8-trimethoxyxanthone. 

## Figures and Tables

**Table 1:** Analysis of the extract by LC–MS.

LC/MS APCI	Peak 1	Peak 2	Peak 3	Peak 4	Peak 5	Peak 6	Peak 7	Peak 8
[M + H]^+^	289	261	289	303	303	275	303	273

**Table 2:** Xanthones content in ethyl acetate extracts of *S. multicaulis* (mg/kg dry matter).

Sample	Peak 1	Peak 2	Peak 3	Peak 4	Peak 5	Peak 6	Peak 7	Peak 8
SM1	1134	5337	1472	1302	4643	316	1957	167
SM2	406	1295	1167	1194	2996	224	3230	146
SM3	913	2600	843	859	2672	260	2230	185
SM4	811	1934	1718	1556	3144	423	2683	224
Average	816	2791	1300	1228	3364	306	2525	181

## Supplementary Materials

^1^H-NMR spectra of isolated chromatographic peaks, list of ^1^H-NMR signals of unassigned xanthones.
