# Supplementary material for: Xanthones Content in Swertia multicaulis D. Don from Nepal
Source: Molecules. 2018 May 3;23(5):1067. doi: 10.3390/molecules23051067 (PMC6102544; doi:10.3390/molecules23051067)
Supplement: Supplementary file 1 [file molecules-23-01067-s001.pdf]

Supplementary Information

Xanthones Content in *Swertia multicaulis* D. Don from Nepal

Binu Timsina[1,2], Pavel Kindlmann[1,2], Maan B. Rokaya[2,3], Naděžda Vrchotová[4], Jan Tříska[4*], Štěpán Horník[5], Jan Sýkora[5]

[1] Institute for Environmental Studies, Faculty of Science, Charles University, Benátská 2, 128 01 Prague, Czech Republic; binu.timsina@gmail.com

[2] Department of Biodiversity Research, Global Change Research Institute, Czech Academy of Sciences, Bělidla 986/4a, 603 00 Brno, Czech Republic; kindlmann.p@czechglobe.cz

[3] Institute of Botany, Czech Academy of Sciences, Zámek 1, 252 43 Průhonice, Czech Republic; rokayamaan@gmail.com

[4] Laboratory of Metabolomics and Isotopic Analyses, Global Change Research Institute, Czech Academy of Sciences, Bělidla 986/4a, 603 00 Brno, Czech Republic; triska.j@czechglobe.cz; vrchotova.n@czechglobe.cz

[5] Institute of Chemical Process Fundamentals, Czech Academy of Sciences, Rozvojová 135, 165 02 Prague, Czech Republic; sykora@icpf.cas.cz; hornik@icpf.cas.cz

* Correspondence: triska.j@czechglobe.cz; Tel.: +420-723-059-668

Contents

Figure S1. ^{1}H-NMR spectrum of decussatin (Peak no. 7) 2

Figure S2. ^{13}C-NMR spectrum of decussatin (Peak no. 7) 3

Figure S3. Full ^{1}H-NMR spectrum of Peak no. 1 and 2 4

Figure S4. Details of the aromatic region of ^{1}H-NMR spectrum of Peak no. 1 and 2 5

Figure S5. Full ^{1}H-NMR spectrum of Peak no. 3 and 4 6

Figure S6. Detail of the aromatic region of ^{1}H-NMR spectrum of Peak no. 3 and 4 7

Figure S7. Full ^{1}H-NMR spectrum of the Peak no. 5 8

Figure S8. Details of the aromatic region of ^{1}H-NMR spectrum of Peak no. 5 9

List of NMR data of unassigned ^{1}H-NMR signals 10

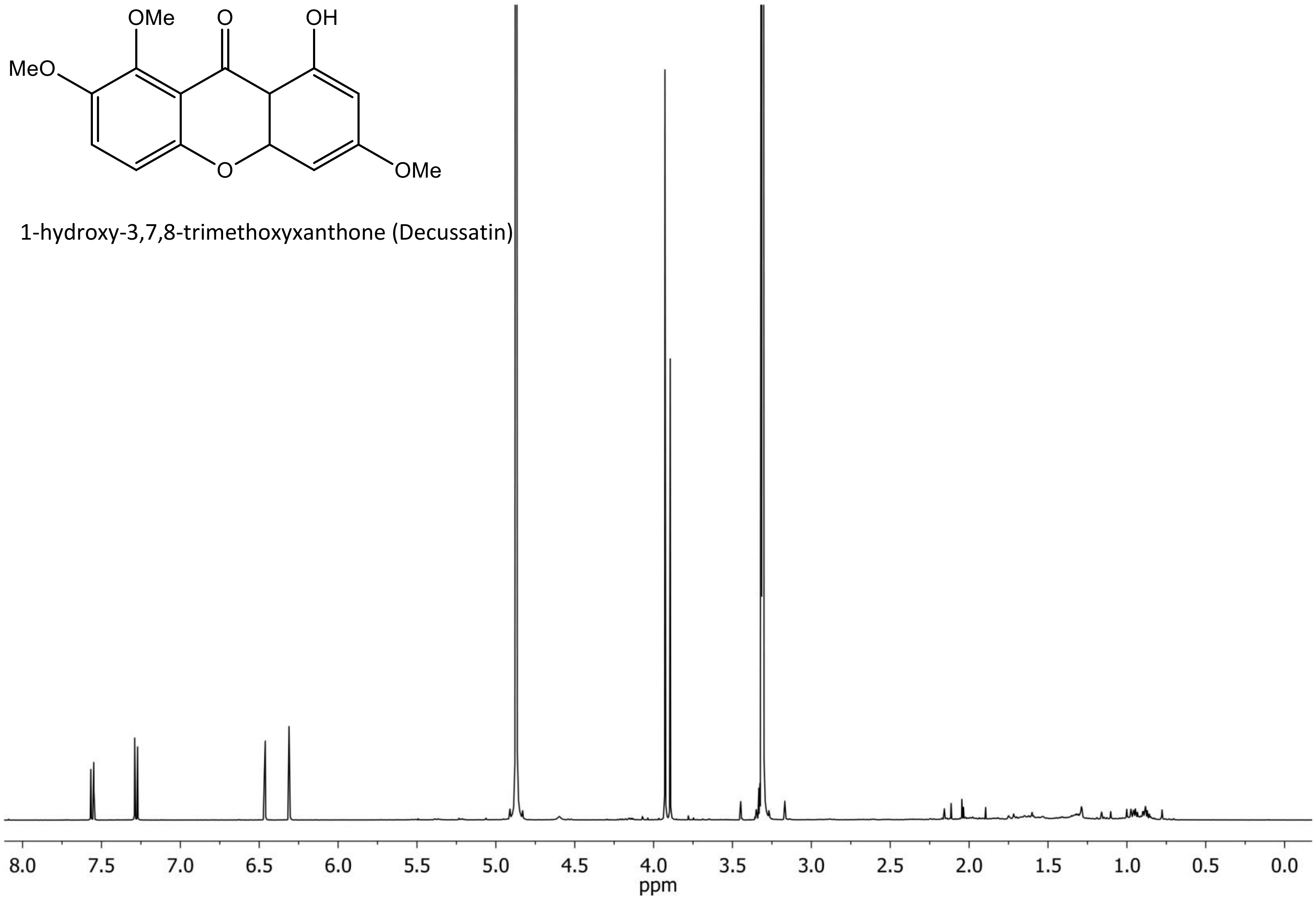

Figure S1. ^{1}H-NMR spectrum of decussatin identified in Peak no. 7 measured in DMSO.

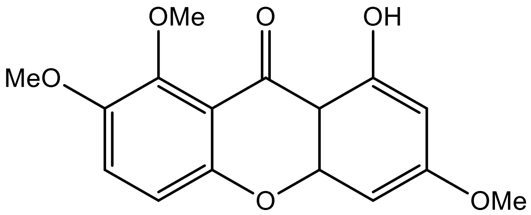

1-hydroxy-3,7,8-trimethoxyxanthone (Decussatin)

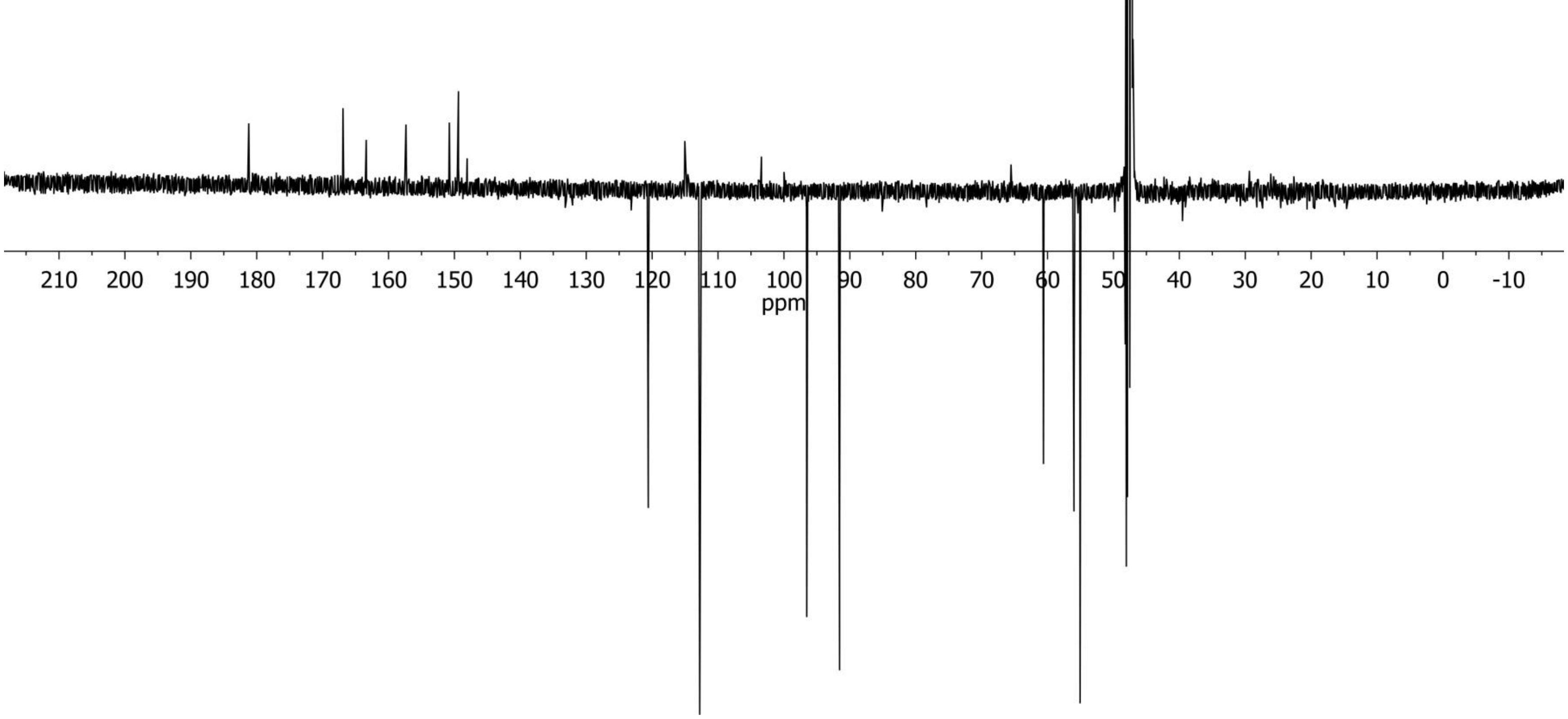

Figure S2. ^{13}C-NMR (APT) spectrum of decussatin identified in peak no. 7 measured in DMSO.

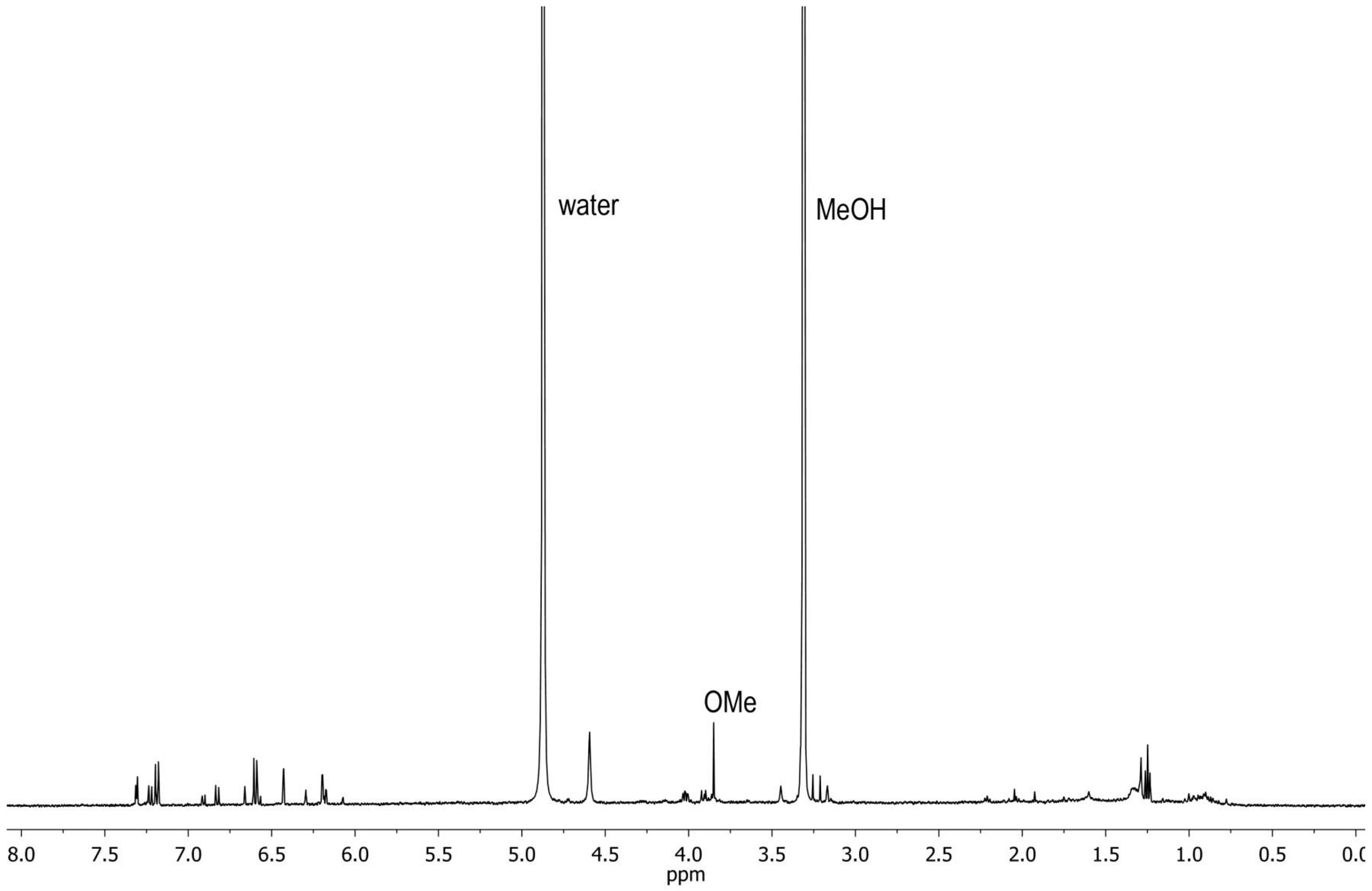

Figure S3. Full ^{1}H-NMR spectrum of the joint fraction of Peak no. 1 and 2 measured in CD_3OD.

#

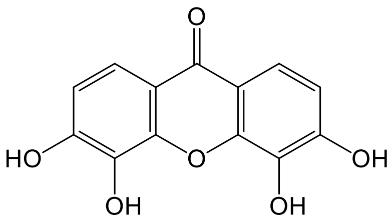

3,3’,4,4’-tetrahydroxy-xanthone

●

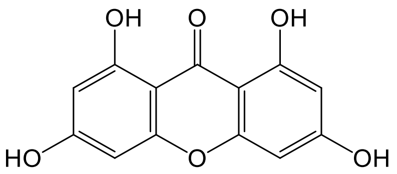

1,1’,3,3’-tetrahydroxy-xanthone

*

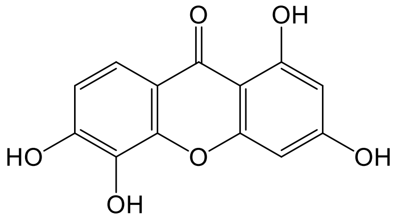

1,3,5,6-tetrahydroxy-xanthone

□

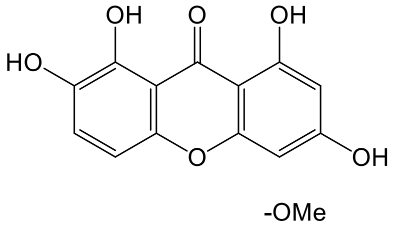

trihydroxy-methoxy-xanthone

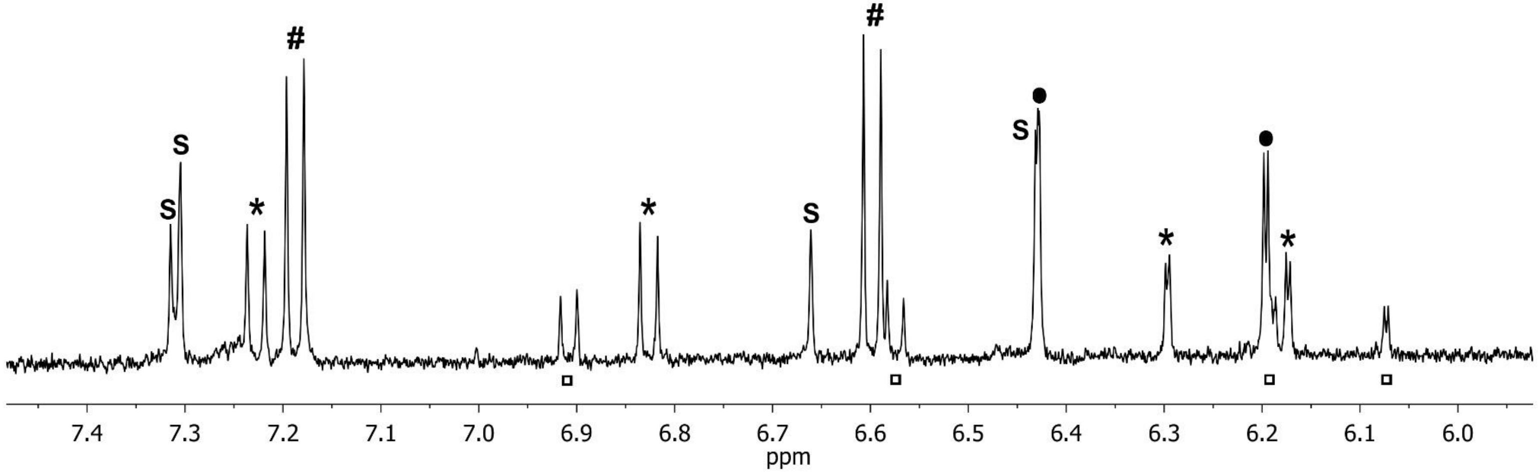

Figure S4. Details of the aromatic region of ^{1}H-NMR spectrum of the joint fraction of Peak no. 1 and 2 measured in CD_3OD with the proposed structures. Four singlets remained unassigned (marked as **S**).

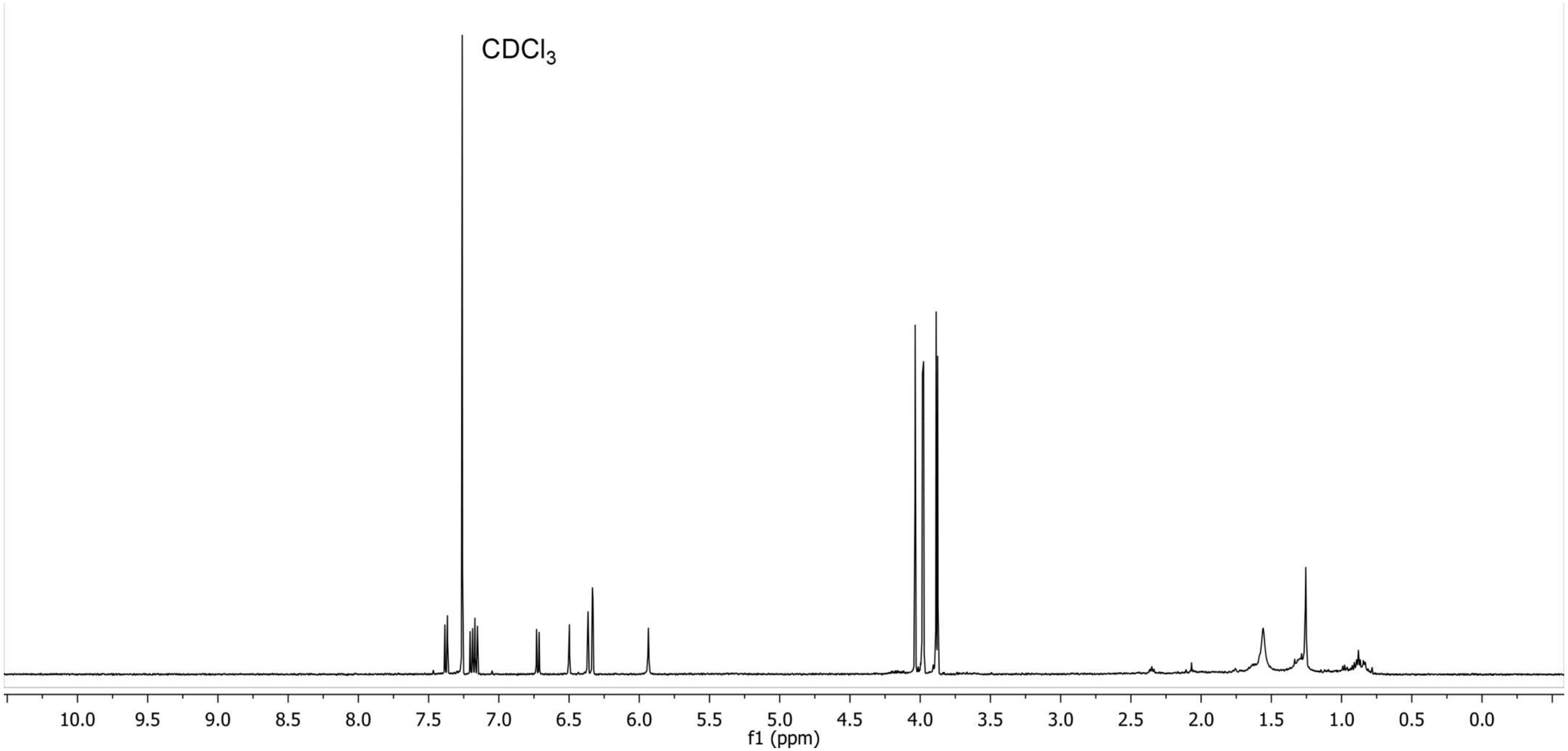

Figure S5. Full ^{1}H-NMR spectrum of the joint fraction of Peak no. 3 and 4 measured in $CDCl_3$.

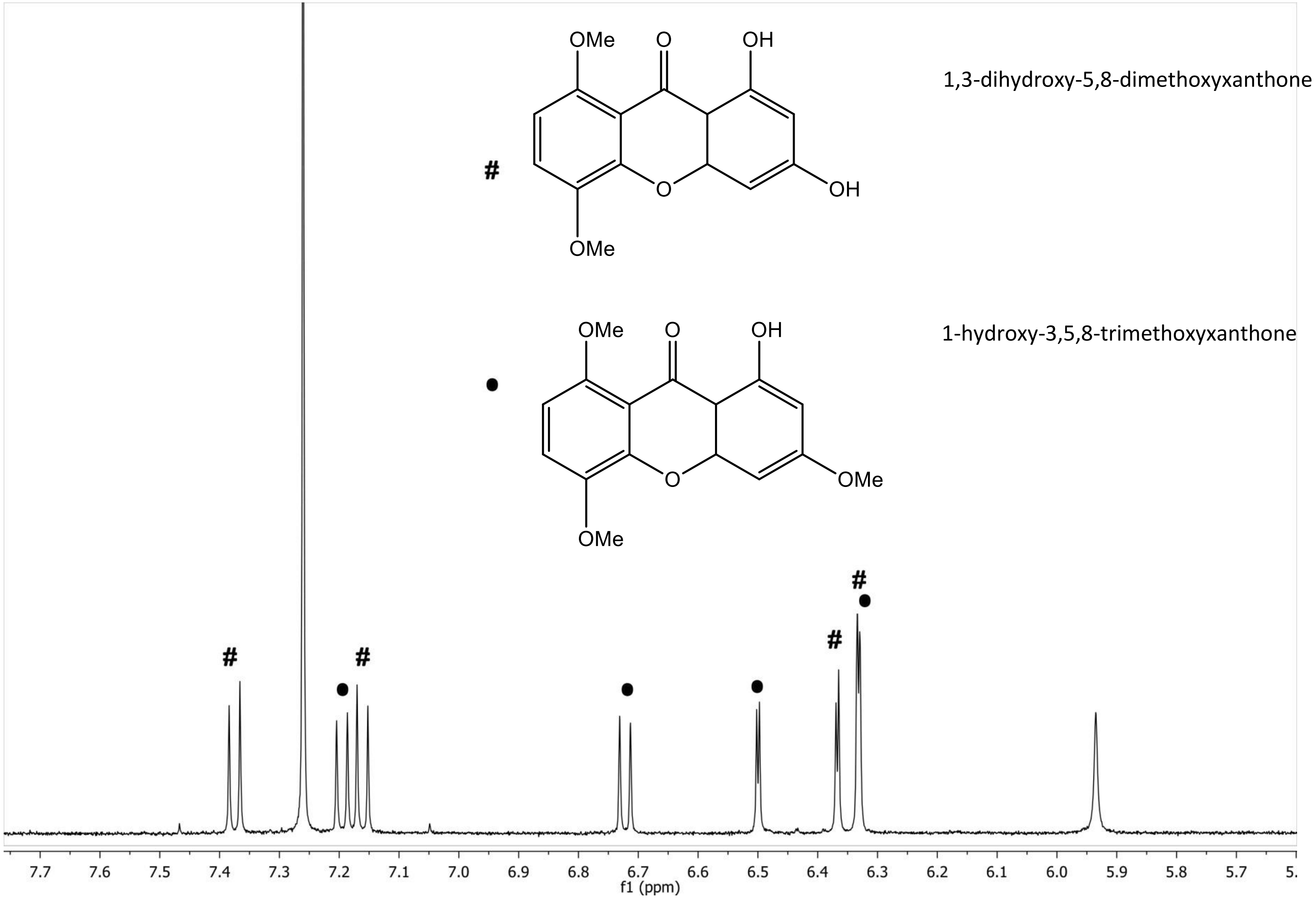

Figure S6. Details of the aromatic region of ^{1}H-NMR spectrum of the joint fraction of Peak no. 3 and 4 measured in $CDCl_3$ with the identified structures.

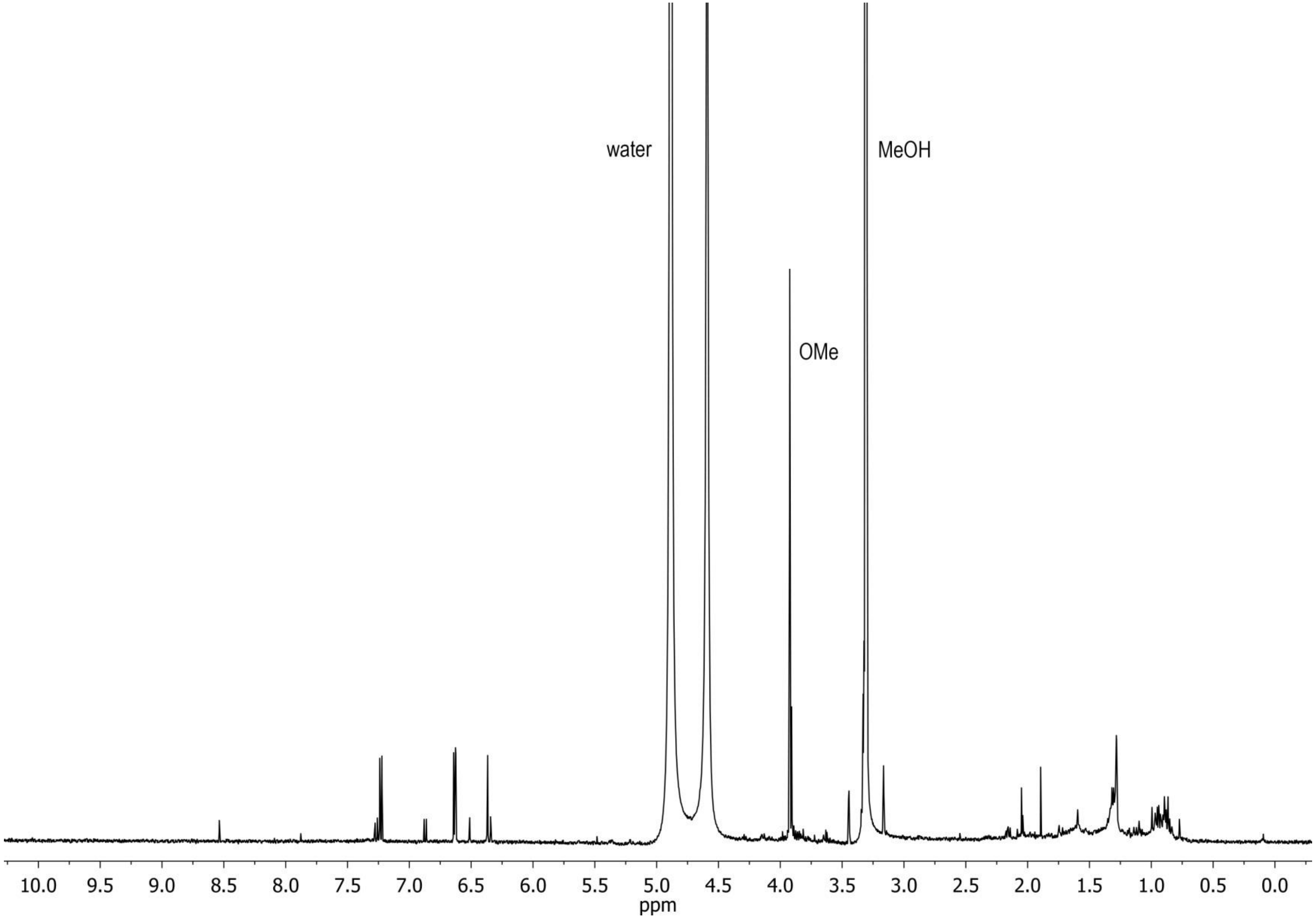

Figure S7. Full ^{1}H-NMR spectrum of the Peak no. 5 measured in CD_3OD.

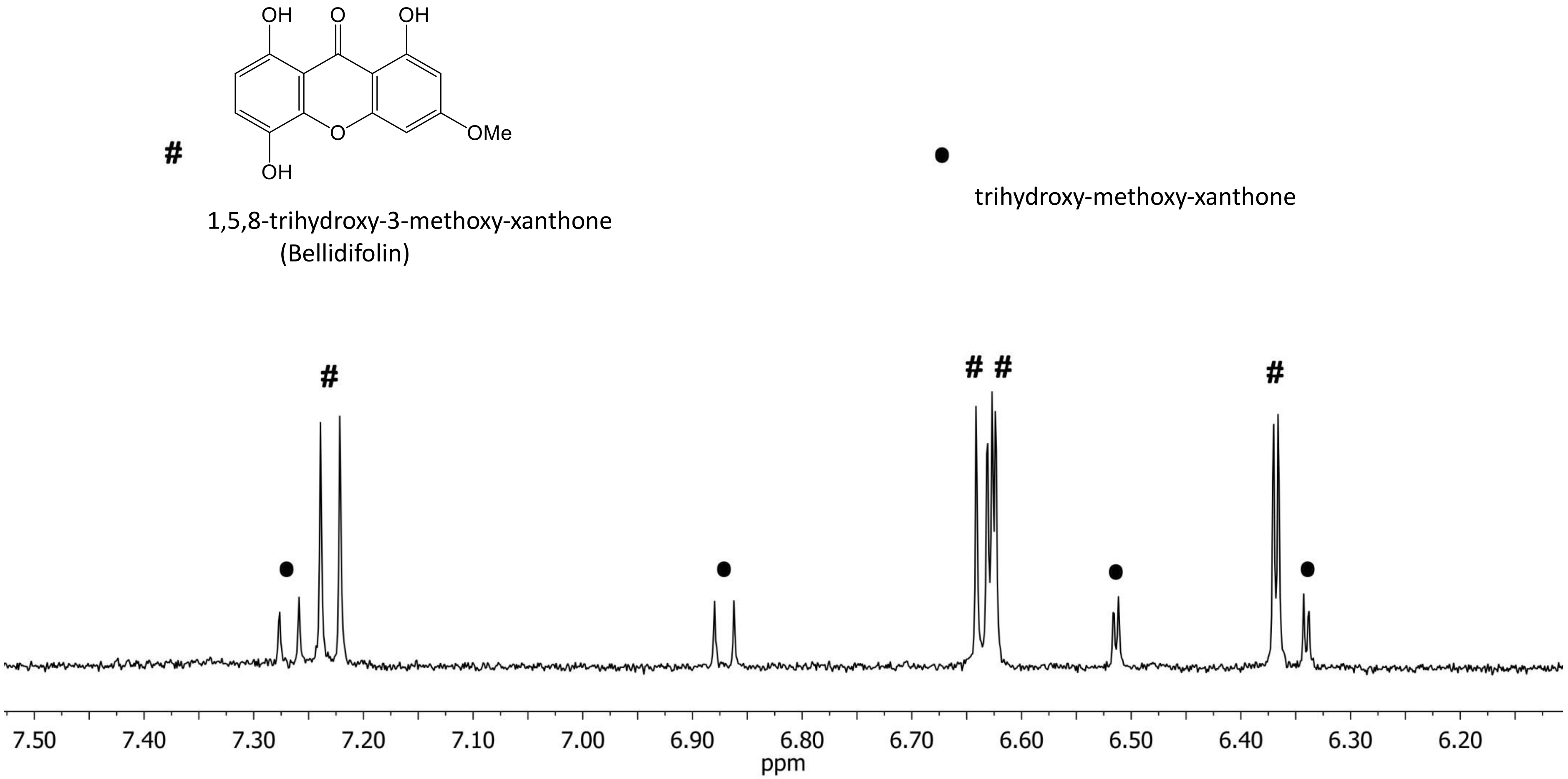

Figure S8. Details of the aromatic region of ^{1}H-NMR spectrum of Peak no. 5 measured in CD_3OD.

The NMR data of merged Peak no. 1 and 2 were collected at 500 MHz NMR spectrometer (Inova500, Varian, Palo Alto, CA, USA) operating at 499.9 MHz for ^{1}H. The trapped chromatographic peaks were evaporated and dissolved in deuterated methanol. Residual signal of methanol was set to 3.31 ppm. The sample concentration allowed only the acquisition of ^{1}H NMR and COSY spectra. The sample contained at least five different compounds. Only one methoxy group was found and it was assigned to one signal set based on the proper ration of the signal integrals. Four singlet signals remained unassigned. The signal intensity of these signals was severely affected by signal overlapping therefore, it was not possible to join the corresponding signals and propose possible compounds.

List of ^{1}H-NMR signals found in merged sample (Peak no. 1 and 2):

Compound **1**: 7.14 (d, 2H, *J* = 8.8 Hz), 6.60 (d, 2H, *J* = 8.8 Hz); proposed structure: 3,3′,4,4′-tetrahydroxyxanthone.

Compound **2**: 6.43 (d, 2H, J = 2.1 Hz), 6.20 (d, 2H, J = 2.1 Hz); proposed structure: 1,1′,3,3′-tetrahydroxyxanthone.

Compound **3**: 7.23 (d, 1H, J = 9.0 Hz), 6.83 (d, 1H, J = 9.0 Hz), 6.30 (d, 1H, J = 2.1 Hz), 6.17 (d, 1H, J = 2.1 Hz); proposed structure: 1,3,5,6-tetrahydroxyxanthone.

Compound **4**: 6.91 (d, 1H, *J* = 8.5 Hz), 6.57 (d, 1H, *J* = 8.5 Hz), 6.19 (d, 1H, *J* = 2.2 Hz), 6.07 (d, 1H, *J* = 2.2 Hz), 3.85 (s, 3H); proposed structure: trihydroxymethoxyxanthone. The location of the methoxy group is not clear. Unassigned singlets 7.31 (s), 7.30 (s), 6.66 (s), 6.42 (s).

Besides bellidifolin, Peak no. 5 contained a minor xanthone compound having also one methoxy group. This isomer of bellidifolin was not identified.

List of ^{1}H-NMR signals found in Peak no. 5:

^{1}H-NMR (CD_3OD, ppm) δ: 7.27 (d, 1H, *J* = 9.0 Hz), 6.87 (d, 1H, *J* = 9.0 Hz), 6.52 (d, 1H, *J* = 2.3 Hz), 6.34 (d, 1H, *J* = 2.3 Hz), 3.91 (s, 3H).
